# Trajectories of delay discounting and smoking from adolescence to young adulthood

**DOI:** 10.1016/j.drugalcdep.2025.112955

**Published:** 2025-11-08

**Authors:** Gezelle Dali, Antoinette Poulton, Tobias Banaschewski, Arun L.W. Bokde, Sylvane Desrivières, Herta Flor, Hugh Garavan, Antoine Grigis, Andreas Heinz, Jean-Luc Martinot, Frauke Nees, Dimitri Papadopoulos Orfanos, Luise Poustka, Michael N. Smolka, Sarah Hohmann, Nilakshi Vaidya, Henrik Walter, Robert Whelan, Gunter Schumann, Robert Hester, the IMAGEN Consortium

**Affiliations:** a Melbourne School of Psychological Sciences, The University of Melbourne, Parkville, VIC 3010, Australia; b German Center for Mental Health (DZPG), Partner Site Mannheim-Heidelberg-Ulm, Germany; c Department of Child and Adolescent Psychiatry and Psychotherapy, Central Institute of Mental Health, Medical Faculty Mannheim, Heidelberg University, Partner site Mannheim-Heidelberg-Ulm, Square J5, Mannheim 68159, Germany; d Discipline of Psychiatry, School of Medicine and Trinity College Institute of Neuroscience, Trinity College Dublin, Dublin, Ireland; e Social, Genetic and Developmental Psychiatry Centre, Institute of Psychiatry, Psychology & Neuroscience, King’s College London, United Kingdom; f Institute of Cognitive and Clinical Neuroscience, Central Institute of Mental Health, Medical Faculty Mannheim, Heidelberg University, Square J5, Mannheim, Germany; g Department of Psychology, School of Social Sciences, University of Mannheim, Mannheim 68131, Germany; h Department of Psychology, University of Vermont, Burlington, Vermont 05405, United States; i NeuroSpin, CEA, Université Paris-Saclay, Gif-sur-Yvette F-91191, France; j German Center for Mental Health (DZPG), Site Tübingen, Germany; k Department of Psychiatry and Psychotherapy, University of Tübingen, Site Tü~bingen, Germany; l Centre Borelli, Gif-sur-Yvette, France; m Institut National de la Santé et de la Recherche Médicale, INSERM U A10 “Trajectoires développementales & psychiatrie”, University Paris-Saclay, Ecole Normale Supérieure Paris-Saclay, CNRS, Gif-sur-Yvette, France; n Institute of Medical Psychology and Medical Sociology, University Medical Center Schleswig Holstein, Kiel University, Kiel, Germany; o Department of Child and Adolescent Psychiatry, Center for Psychosocial Medicine, University Hospital Heidelberg, Heidelberg, Germany; p Department of Psychiatry and Psychotherapy, Technische Universität Dresden, Dresden, Germany; q Department of Child and Adolescent Psychiatry and Psychotherapy, University Medical Center Hamburg-Eppendorf, Hamburg, Germany; r Centre for Population Neuroscience and Stratified Medicine (PONS), Department of Psychiatry and Psychotherapy, Charité Universitätsmedizin Berlin, Germany; s Department of Psychiatry and Psychotherapy CCM, Charité – Universitätsmedizin Berlin, corporate member of Freie Universität Berlin, Humboldt-Universität zu Berlin, and Berlin Institute of Health, Berlin, Germany; t School of Psychology and Global Brain Health Institute, Trinity College Dublin, Ireland; u Centre for Population Neuroscience and Precision Medicine (PONS), Institute for Science and Technology of Brain-inspired Intelligence (ISTBI), Fudan University, Shanghai, China

**Keywords:** Adolescent, Delay discounting, Impulsivity, Nicotine, Reward sensitivity, Smoking

## Abstract

**Background:**

Delay discounting is consistently implicated in nicotine use, with individuals dependent on smoking exhibiting greater discounting rates than those who do not smoke. The temporal relationship of delay discounting and smoking, however, has been relatively understudied as much of the existing literature has used cross-sectional designs. This study examined whether delay discounting is predictive of both the initiation of occasional smoking and the transition from occasional to daily use and whether escalating smoking behaviour predicts increased delay discounting.

**Methods:**

Participants were drawn from the IMAGEN sample – a large, longitudinal, multicentre study. Data were collected at ages 14, 16, 18 and 22 years. Our sample consisted of 1668 participants (52 % female) who had completed at least two waves of data collection. Delay discounting was measured using the 27-item Monetary Choice Questionnaire. The European School Survey Project on Alcohol and Drugs (ESPAD) and the Timeline Follow-back were used to assess smoking behaviours.

**Results:**

Higher delay discounting predicted a greater likelihood of initiation of occasional use but not the transition to daily smoking. The trajectory of smoking frequency was predicted by both baseline levels of delay discounting and the trend of delay discounting over time. Smoking, however, was not found to predict changes in delay discounting.

**Conclusions:**

High delay discounting appears to precede the initiation of smoking and is predictive of the trajectory of smoking but may not distinguish between discrete states of smoking. Identifying heightened delay discounting in young people may offer the opportunity to prevent excessive smoking trajectories before they are initiated.

## 1. Introduction

Higher impulsive decision-making confers the propensity to favour immediate reward irrespective of delayed outcomes and is implicated in several psychiatric conditions, including substance use disorders (Amlung et al., 2019; Bickel et al., 2019; Fernie et al., 2010; MacKillop et al., 2011). In the context of substance use, impulsive decision-making manifests as increased sensitivity to the immediate rewarding aspects of the drug, such as euphoria and relief from symptoms of withdrawal, and insensitivity to the negative outcomes such as deleterious health and social consequences which tend to have a gradual and delayed onset (MacKillop et al., 2011; Poulton and Hester, 2020). One common approach to assess impulsive decision-making is through the use of discounting tasks, which quantify the rate at which future rewards are devalued as a function of delay (Field et al., 2007; Kirby et al., 1999; Rung and Madden, 2018). A higher discounting rate indicates a preference for smaller, immediate rewards at the expense of larger rewards later, and is therefore indicative of greater impulsive decision-making (Wittmann and Paulus, 2008). This pattern of responding is prototypic of individuals with addictive disorders and has led to extensive research on the role of delay discounting in the aetiology and maintenance of substance use disorders.

The substance use literature has demonstrated robust relationships between delay discounting and nicotine dependence. That is, individuals with smoking dependence have consistently been reported to discount rewards more steeply than individuals who do not smoke; a finding that has been observed with varying reward magnitudes and commodities (Baker et al., 2003; Bickel et al., 1999; Bickel et al., 2008; Businelle et al., 2010; Friedel et al., 2014; Hofmeyr et al., 2017; MacKillop et al., 2011; Mitchell and Wilson, 2012; Reynolds, 2004, 2006; Reynolds and Fields, 2012; Reynolds et al., 2004). These results have also been replicated in more recent work comparing delay discounting between participants who smoke and those that do not across different smoking modalities (i.e., e-cigarettes, combustible cigarettes) (Białaszek et al., 2017; DeHart et al., 2020). Findings on whether delay discounting differs as a function of nicotine dependence are, however, equivocal. While there is evidence of continuous associations between delay discounting and nicotine dependence severity (Amlung and MacKillop, 2014; Amlung et al., 2017), research examining delay discounting across smoking states has yielded mixed results. For example, participants who smoked lightly were found to display greater delay discounting than participants who did not smoke, while no differences were observed between participants who smoked a light amount and those that smoked heavily (Johnson et al., 2007). Similarly, adolescents experimenting with smoking have been found to discount more than individuals who do not smoke but not individuals who smoke daily (Reynolds and Fields, 2012). In contrast, individuals with smoking dependence have been reported to display greater delay discounting than individuals who have never smoked, smoke occasionally or formally smoked, with no significant differences between the latter three groups (Sweitzer et al., 2008). In other studies, this absence of difference between participants who do not smoke and those that smoke occasionally was only observed for actual, and not hypothetical, monetary rewards (Heyman and Gibb, 2006). Taken together, these mixed findings raise the question of whether heightened delay discounting precedes nicotine dependence and remains stable with escalating smoking behaviour or whether delay discounting and smoking reciprocally influence one another over time resulting in increased nicotine dependence.

Clarifying whether delay discounting offers a predictive index of both the initiation of light or occasional use and the transition to dependence is critical as research has shown that 67 % of individuals who try cigarettes eventually transition to dependence (Lopez-Quintero et al., 2011). That is, it is important to determine whether delay discounting can differentiate individuals who transition from occasional to daily use from those who cease smoking because of its potential impact on when, and whom, to target interventions. Few studies though have elucidated the temporal relationship of delay discounting and nicotine dependence as much of the literature has relied on cross-sectional designs which preclude causal inferences. Addressing this in a six-year prospective longitudinal study of 947 adolescents, Audrain-McGovern et al. (2009) found that increases in baseline delay discounting predicted an increased likelihood of smoking uptake and that smoking did not have a significant effect on delay discounting. Moreover, while delay discounting was higher in the smoking versus non-smoking trajectory, delay discounting did not discriminate between the smoking trajectories (i.e., early/fast adopters and slow smoking progressors). Based on these findings, the authors posited that delay discounting may offer an index by which to screen for smoking vulnerability but cannot predict the pattern of smoking acquisition. These findings are consistent with earlier reports that return to smoking following abstinence was predicted by baseline delay discounting and that delay discounting remained stable over time, suggesting that premorbid delay discounting also predicts success of cessation attempts (Yoon et al., 2007). It is worth noting though that findings of stable delay discounting somewhat juxtapose studies which have examined delay discounting over the lifespan and report that patterns of delay discounting change across developmental periods (Lu et al., 2023). Further, while Audrain-McGovern et al. (2009) examined differences in smoking trajectories related to the speed of uptake, they did not explore whether delay discounting is a marker of transition between smoking states (e.g., from occasional use to daily use). To date, however, Audrain-McGovern et al. (2009) remains the only prospective study on the predictive utility of delay discounting in the acquisition and progression of smoking. Given the dearth of research and the presence of conflicting results, further longitudinal work is required to clarify the role of delay discounting in nicotine use, particularly with relation to transition from occasional use to dependence.

The current study forms the largest longitudinal investigation into the role of delay discounting in smoking from mid-adolescence to early adulthood. This study aimed to determine whether delay discounting is predictive of both the initiation of smoking and the transition from occasional to daily use. As a secondary aim, we sought to clarify whether escalating smoking behaviour over time predicts increased delay discounting. It is expected that an improved understanding of the link between delay discounting and smoking will facilitate the development of prevention and intervention strategies to reduce the health and economic impact of smoking. We hypothesised that delay discounting would predict the initiation of smoking (i.e., the transition from never to occasional use) but would not be predictive of the transition from occasional to daily use. It was also hypothesised that overall smoking trends would not influence delay discounting rates over time.

## 2. Methods

### 2.1. Participants

Participants in the current study were drawn from the IMAGEN sample – a large, longitudinal, multicentre study (Schumann et al., 2010). Participants were recruited from eight sites across France, Ireland, England and Germany. Data were collected at ages 14, 16, 18 and 22 and comprised a comprehensive assessment of adolescent substance use and decision-making. Local ethics research committees approved the study at each site (London, England: Psychiatry, Nursing and Midwifery Research Ethics Subcommittee, Waterloo Campus, King’s College London; Nottingham, England: University of Nottingham Medical School Ethics Committee; Mannheim, Germany: Medizinische Fakultaet Mannheim, Ruprecht Karl Universitaet Heidelberg and Ethik-Kommission II an der Fakultaet fuer Kliniksche Medizin Mannheim; Dresden, Germany: Ethikkommission der Medizinischen Fakultaet Carl Gustav Carus, TU Dresden Medizinische Fakultaet; Hamburg, Germany: Ethics Board, Hamburg Chamber of Physicians; Paris, France: CPP IDF VII (Comité de protection des personnes Ile de France), ID RCB: 2007-A00778-45 September 24, 2007; Dublin, Ireland: TCD School of Psychology REC; and Berlin, Germany: Ethics Committee of the Faculty of Psychology). Written consent was obtained from the adolescent’s parent or guardian, and verbal assent was obtained from the adolescent. Data from 1668 participants (52 % female) who had completed at least two waves were included in the current study.

### 2.2. Delay Discounting

The Monetary Choice Questionnaire (MCQ) was administered at each wave to assess delay discounting. The MCQ is a 27-item measure that requires participants to make hypothetical choices between monetary rewards available immediately and larger rewards available after a delay (Kirby et al., 1999). Individual discounting rate, k, is derived from the following hyperbolic formula (Mazur, 1987), where V represents the present value of the delay reward A at delay D:

$$V=\frac{A}{1\;+\;kD}$$

Larger k-values indicate greater discounting of the delayed reward. In line with Kaplan et al. (2016), consistency scores were determined for data collected at each wave timepoint. Timepoints with a consistency score less than 75 % were excluded from analysis. Forty-four data points were removed which resulted in the exclusion of eight participants because no MCQ data was remaining for these participants. As is standard practice, k-values were natural-log transformed prior to analyses.

### 2.3. Smoking states

The European School Survey Project on Alcohol and Drugs (ESPAD; Hibell et al., 1997) was administered at each wave to obtain measures of age of onset and quantity and frequency of alcohol and illicit drug use in one’s lifetime, past 12 months, past 30 days and past week. Responses to the following smoking questions were used to classify participants’ smoking states:

“On how many occasions (if any) during your lifetime have you smoked cigarettes?”

“How frequently have you smoked cigarettes during the LAST 30 DAYS?”

Each question requires participants to select a response from seven ordinal options. For lifetime occasions, the response options are: 0 occasions; 1–2 occasions; 3–5 occasions; 6–9 occasions; 10–19 occasions; 20–39 occasions; 40 or more occasions. For use in the last 30 days, the options are: ‘Not at all’; ‘Less than 1 cigarette per week’; ‘Less than 1 cigarette per day’; ‘1–5 cigarettes per day’; ‘6–10 cigarettes per day’; ‘11–20 cigarettes per day’; ‘More than 20 cigarettes per day.’ Using responses to these questions, smoking states were defined at each wave. Participants were classified as ‘*never*’ if they reported having never smoked cigarettes in their lifetime. Participants were classified as ‘*non-current*’ if they had reported smoking cigarettes in their lifetime or at a previous wave but did not report any use in the last 30 days. Participants were classified as ‘*occasional*’ if they reported ‘Less than 1 cigarette per week’ or ‘Less than 1 cigarette per day’ in the last 30 days. Participants were classified as ‘*daily*’ if they selected any category that endorsed daily cigarette use (i.e., ‘1–5 cigarettes per day’, ‘6–10 cigarettes per day’, etc.) for the last 30 days.

### 2.4. Smoking frequency

The Timeline Follow-back (TLFB) (Sobell and Sobell, 1973) is a retrospective diary method which was used to derive total smoking frequency over the preceding 30-days. The TLFB was administered at each wave with the exception of the second wave.

### 2.5. Data analysis

#### 2.5.1. Multistate model

A multistate, continuous time Markov model was used to estimate the transition probability between the smoking states (i.e., never, non-current, occasional, and daily) using the *msm* package (Jackson, 2011) in R (R Core Team, 2021). Multistate models represent discrete states that change through time but are intermittently observed. That is, the model allows transitions between states to occur at unknown times between observation timepoints. Multistate modelling is a common analytical method for modelling transitions between smoking states (Johnson et al., 2021; Mayet et al., 2011; Parnham et al., 2024).

Continuous-time Markov models are defined by transition intensities that govern these movements between states. The transition intensity, $q_{rs}(t,\;z(t))$, represents the instantaneous risk of moving from one state, r, to another state, s:

$$q_{rs}(t,\;z(t))=\underset{\delta t\to 0}{lim}P(S(t+\delta t)=s|S(t)=r)/\delta t$$

The intensities depend on the time of the process, t, as well as individual-specific or time-varying covariates, z(t)). The transition intensities, $q_{rs}$, form a matrix Q whose rows sum to zero. The transition probability matrix over any time unit can be computed as the matrix exponential: *P*(*t*) = *Exp*(*tQ*). The multistate model also estimates the mean sojourn time in each state, which is the average period a participant spends in a transient state before moving to another state. The expected sojourn time is calculated as: − 1/*q*_*rr*_.

**Fig. 1.**
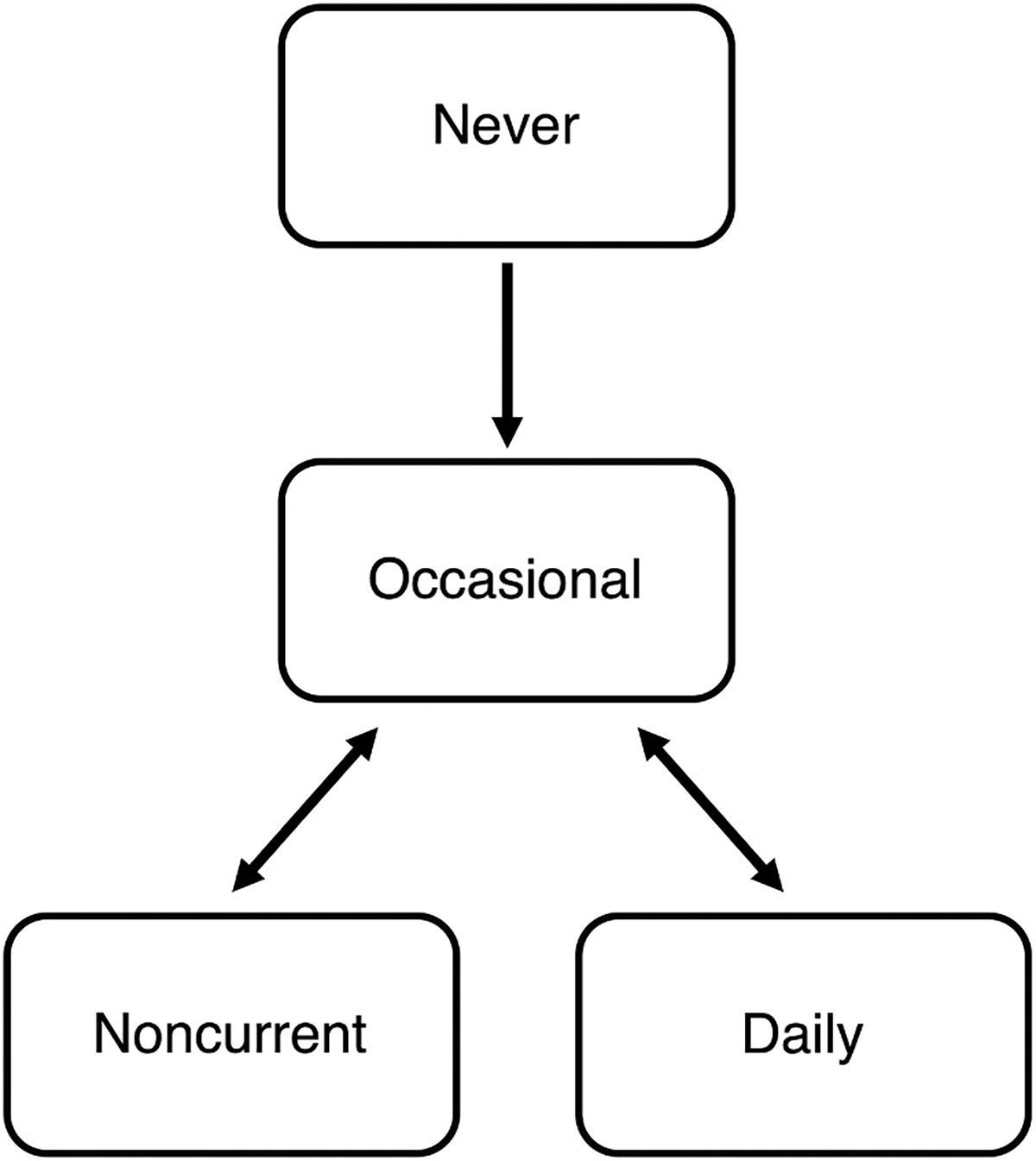
Permitted direct transitions between smoking states. Smoking state was modelled as a quantity that changes smoothly over time and with an ordered state of severity. For example, participants classified as ‘*never*’ will have to go through ‘*occasional*’ to reach ‘*daily*’ at an adjacent wave. Thus, we did not permit direct transitions from ‘*never*’ to ‘*daily*’, ‘*non-current*’ to ‘*daily*’, and ‘*daily*’ to ‘*non-current*’. The model still permits these transitions for any discrete time unit (e.g., from one day to the next).

Permitted transitions for our model are displayed in Fig. 1. Since individuals tend to move through intermittent or occasional smoking before reaching daily use (Lopez-Quintero et al., 2011), we modelled smoking state as representing a quantity that changes smoothly over time and with an ordered state of severity. For example, participants classified as ‘*never*’ would have to go through ‘*occasional*’ to reach ‘*daily*’ at an adjacent wave. Therefore, we did not permit direct transitions from ‘*never*’ to ‘*daily*’, ‘*non-current*’ to ‘*daily*’, and ‘*daily*’ to ‘*non-current*’. The model still permitted these transitions for any discrete time unit (e.g., from one day to the next). Time for each participant started at Wave 1 (time 0) and each subsequent wave time was determined by the number of years that had elapsed since Wave 1. To determine the effect of delay discounting on transition between states, discounting rate, *In*(*k*), at each timepoint was included as a covariate. We considered the inclusion of age (in years) and sex as covariates as prior work has demonstrated an association between these variables and smoking initiation and transition to dependence (Huggett et al., 2019; Okoli et al., 2013), as well as recruitment site since smoking culture, policies and norms may differ across recruitment locations. The best fitting multistate model was based on evaluation of Akaike’s information criterion (Jackson et al., 2022).

#### 2.5.2. Latent growth model

**Fig. 2.**
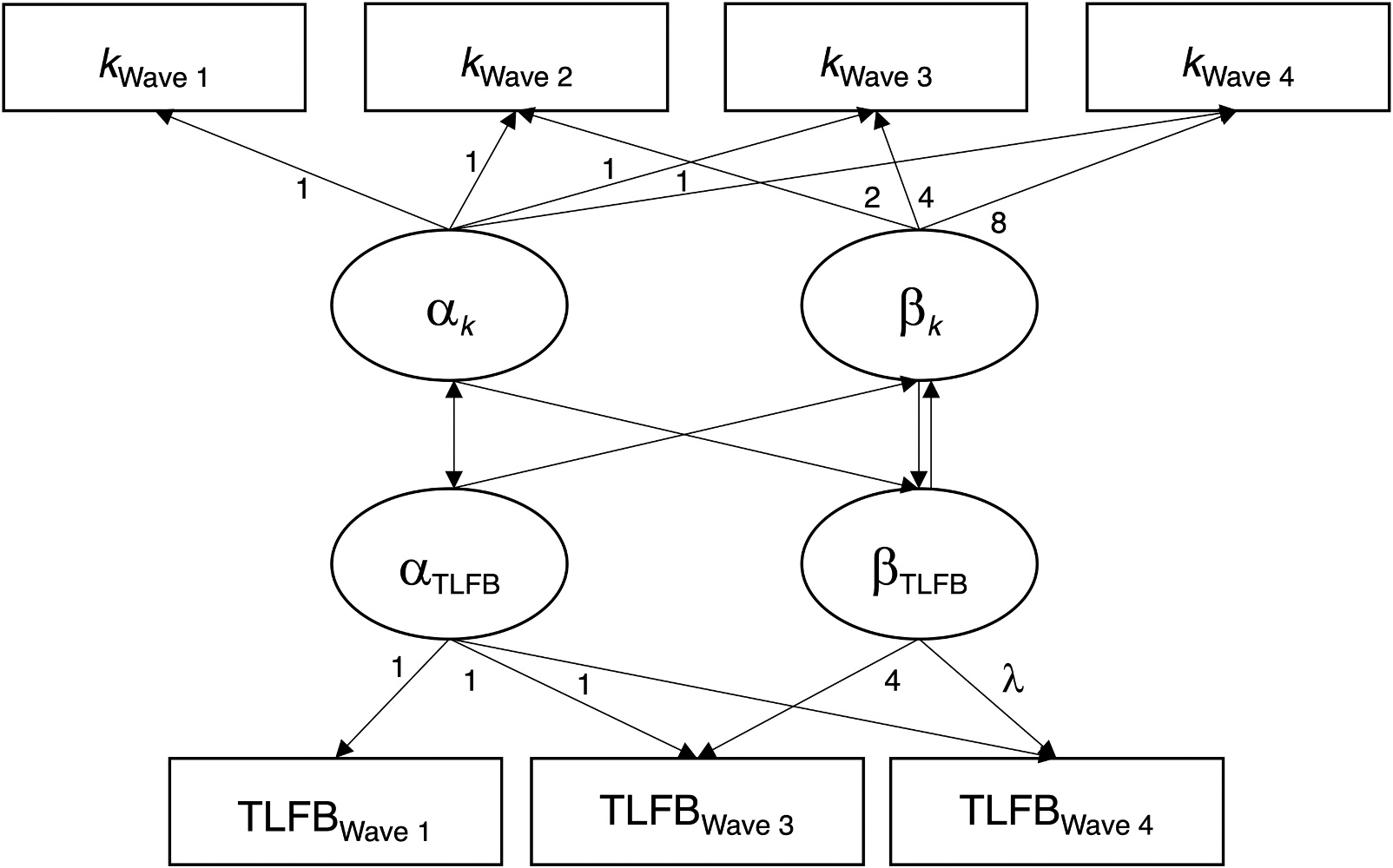
Bivariate latent growth curve model for delay discounting and smoking frequency. Rectangles represent observed variables and ovals represent latent variables. Single-headed arrows represent regression paths and double-headed arrows represent covariance paths. For simplicity, the sex covariate is not displayed. α = intercept; β = slope; λ = free slope; TLFB = Timeline Follow-back; *k* = discounting rate.

To determine the bidirectional relationship between the development of delay discounting and smoking over time, we computed a bivariate latent curve growth model (Fig. 2) using the *lavaan* package in R (Rosseel, 2012). Latent growth curve models estimate the trajectory of change for constructs by modelling individual differences in growth patterns across multiple time points (Duncan and Duncan, 2009). This model allowed us to assess whether smoking trends influence delay discounting rates over time. For both smoking and delay discounting, two latent growth factors were modelled: an intercept factor which captured the baseline level of the construct and a slope factor which represented the trajectory of that construct over time. Latent growth factors for smoking were estimated using TLFB smoking frequency and latent growth factors for delay discounting were estimated using *In*(*k*). To determine the reciprocal influences between baseline and trend levels of smoking and delay discounting over time, we regressed the slope of delay discounting onto the intercept and slope of smoking and vice versa. Sex was included as a covariate, and intercepts were permitted to covary. Model parameters were estimated using robust maximum likelihood and full information maximum likelihood was used to account for missing values. Model fit was estimated using the following standard fit indices: comparative fit index (CFI), root mean square error of approximation (RMSEA) and the standardised root mean square residual (SRMR). Per recommendations from Hu and and Bentler (1999), criteria for good model fit are CFI > .95, RMSEA < .05, and SRMR < .08. For reference, we also report the chi-squared goodness-of-fit test. It should be noted though that this test has been shown to be sensitive to large sample sizes, typically resulting in model rejection despite irrelevant discrepancy between the sample and model-implied covariance matrix (Schermelleh-Engel et al., 2003).

## 3. Results

### 3.1. Descriptive statistics

Descriptive statistics for sample characteristics are presented in Table 1. At the first wave, 75 % of participants had never smoked and only 2 % of participants reported smoking daily. By the fourth wave, 24 % of participants had never smoked and 20 % were daily smokers. Frequencies of transitions between smoking states are presented in Supplementary Material (Table 1S).

### 3.2. Multistate model

The transition probability matrix estimates the cumulative likelihood that an individual in one smoking state will transition to a different state or remain in the same state in an 8-year period (Table 2). The probability of retention in the same state was highest for daily smoking (50 %, 95 % CI = 43–57 %) and lowest for occasional smoking (21 %, 95 % CI = 19–23 %). Participants who smoked occasionally were more likely to transition to non-current use (44 %, 95 % CI = 41–47 %) than daily use (35 %, 95 % CI = 31–39 %). Mean sojourn times (Supplementary Table 2S) indicated that participants spend the most time in daily use prior to transition (7.01 years, 95 % CI = 5.39–9.25). By contrast, time spent in occasional use before transition was estimated to be 0.08 years, 95 % CI = 0.01–0.44.

Hazard ratios for the effects of delay discounting and age on each transition are presented in Table 3. Delay discounting was significantly associated with transition risk from never to occasional smoking, HR = 1.07, 95 % CI [1.02, 1.12], and from non-current to occasional smoking, HR = 1.59, 95 % CI [1.03, 2.46]. That is, a unit increase in delay discounting rate was associated with a 7 % increase in the likelihood of transitioning from never to occasional smoking, and a 59 % chance of transitioning from non-current to occasional smoking. There was no effect of delay discounting on the transition from occasional to daily smoking, HR = 1.09, 95 % CI [0.99, 1.21]. With the exception of daily smoking, older age was significantly associated with a reduced likelihood of transitioning out of each state. Females were more likely than males to transition from daily to occasional smoking, HR = 1.57, 95 % CI [1.06, 2.33]. There was no effect of sex on any other transition.

### 3.3. Latent growth curve model

The latent growth curve model exhibited good fit, CFI = .97, RMSEA = .05, 95 % CI [.04,.06], SRMR = .03, and χ^2^(17) = 97.0, *p* < .001. The model demonstrated that the average delay discounting trend (i.e., the intercept of the slope, β_In(k)_) was non-significant, γ_βIn(k)_ = 2.50, 95 % CI [−1.78, 6.78], indicating no evidence of change in delay discounting from baseline. In line with this, there was no effect of baseline smoking frequency, β = −1.90, 95 % CI [−6.69, 2.89], or the trend of smoking over time on the trend of delay discounting, β = −4.73, 95 % CI [−14.28, 4.83]. Removal of the delay discounting slope and its associated regression paths resulted in a significant reduction in model fit, Δχ^2^ = 30.9, Δdf = 3, *p* < .001. Therefore, we opted to retain the original model. The average smoking trend (i.e., the intercept of the slope, β_TLFB_) was found to be significant, γ_βTLFB_ = 3.09, 95 % CI [1.50, 4.67], indicating increased smoking frequency over time. A significant positive correlation was found between baseline delay discounting and baseline smoking, *r* = .24, 95 % CI [0.16, 0.33]. Baseline delay discounting had a significant positive effect on smoking trend, β = 0.67, 95 % CI [0.21, 1.13]. That is, a higher initial delay discounting rate predicted greater increases in smoking frequency from mid-adolescence to early adulthood. Further, the delay discounting trend was found to significantly predict the smoking trend, β = 2.04, 95 % CI [0.51, 3.56], such that increases in delay discounting over time predicted increased smoking frequency. Full output of the latent growth model is presented in Supplementary Material (Table 3S).

## 4. Discussion

The current study forms the largest longitudinal investigation into the role of delay discounting in the initiation of smoking and the transition from occasional to daily use. This study also examined the reciprocal relationship between delay discounting and smoking to ascertain whether delay discounting is influenced by increased smoking behaviour. Consistent with our hypotheses, findings indicated that higher delay discounting predicted a greater likelihood of initiation of occasional use; however, there was no effect of delay discounting on likelihood of transition to daily smoking. Results of our latent growth model demonstrated that the trend of smoking was predicted by both baseline levels of delay discounting and the trajectory of delay discounting over time. Smoking, however, was not found to have a predictive effect on delay discounting. Thus, greater delay discounting appears to precede the initiation of smoking and is not influenced by escalating smoking behaviour.

When modelling the transition between discrete smoking states (i.e., never, non-current, occasional and daily), our results revealed a small positive predictive effect of delay discounting on transition from never smoking to occasional use, with a non-significant effect on the transition from occasional to daily smoking. This pattern of findings was largely consistent with our latent growth model, where both baseline delay discounting and changes in delay discounting over time predicted the overall trend in smoking frequency. These results align with Audrain-McGovern et al. (2009) who found that baseline delay discounting predicted overall smoking trajectories but did not differentiate between discrete categories of smoking. Taken together, delay discounting may play a more significant role in the early stages of smoking uptake and may be more indicative of overall smoking status or long-term patterns rather than specific transitional stages. That is, delay discounting may exert a gradual influence on smoking escalation. As such, differences in delay discounting between individuals with occasional and daily use may be less discernible, particularly when severity of use is moderate– as was the case in the current sample, where most participants who smoked daily smoked fewer than 10 cigarettes per day. Indeed, the cross-sectional literature has provided robust evidence of continuous associations between delay discounting and nicotine dependence severity (Amlung and MacKillop, 2014; Amlung et al., 2017), while differences between smoking states (i.e., intermittent and daily smoking) remain largely equivocal (Heyman and Gibb, 2006; Johnson et al., 2007; Reynolds and Fields, 2012; Sweitzer et al., 2008). The current study supports the notion that delay discounting may reflect vulnerability to sustained smoking rather than serving as a marker which differentiates occasional from daily smoking.

Delay discounting in the current study was not found to be influenced by either baseline levels of smoking or changes in smoking behaviour. This aligns with Audrain-McGovern et al. (2009) who likewise reported no effect of smoking on the trend of delay discounting during mid-adolescence to early adulthood. Our findings counter prior cross-sectional studies which suggest that greater delay discounting in individuals dependent on smoking relative to individuals who smoke lightly or smoked previously may be due to increased nicotine exposure (Bickel et al., 1999; Yi et al., 2008). While we do not discount the possibility that delay discounting may have been influenced by contextual factors not assessed in this study (Acuff et al., 2023), such as socioeconomic circumstances (Peviani et al., 2024), the absence of change in delay discounting over time suggests that high delay discounting may reflect an early risk factor for the progression of nicotine use rather than emerging as a consequence of smoking behaviour. Therefore, previous findings suggesting that individuals who currently smoke, but not those who formerly smoked, discount rewards more steeply than individuals who have never smoked (Sweitzer et al., 2008) may indicate that individuals with relatively lower delay discounting rates are more likely to successfully quit smoking. While we did find a predictive effect of delay discounting on the transition from non-current to occasional use, the short sojourn times in these categories suggest that participants classified as non-current were unlikely to represent true cessation attempts. Instead, their status likely reflects the fluctuations in drug use common during adolescence and early adulthood (Mistry et al., 2015). When considering existing work in treatment-seeking individuals, however, there is indeed evidence that return to smoking can be predicted by delay discounting (Syan et al., 2021). Taken together, delay discounting does not appear to be malleable in the context of changes in nicotine exposure and rather may present as an early risk factor which predicts future smoking behaviour.

The current results have implications for understanding the significance of delay discounting in smoking and substance use more broadly. Our findings align with longitudinal work demonstrating that higher delay discounting predicts the development of alcohol, cocaine and poly-substance use (Felton et al., 2020; Fernie et al., 2013; Fröhner et al., 2022; Kim-Spoon et al., 2019; Kräplin et al., 2020), with no evidence that use alters delay discounting (Felton et al., 2020; Fernie et al., 2013; Fröhner et al., 2022). Taken together, our results lend support to the use of delay discounting as a potential transdiagnostic marker of substance use (Amlung et al., 2019). In the context of smoking, our findings suggest that higher delay discounting may increase vulnerability to both smoking initiation and escalation in adolescence and young adulthood. High delay discounting may serve as a behavioural process in smoking progression by promoting individuals to seek out the immediate rewarding aspects of nicotine over temporally distal events such as better health and relationships (Johnson et al., 2007). The current results suggest potential utility for delay discounting as one possible marker within a broader risk assessment framework to help identify adolescents and young adults who may be more likely to initiate smoking. This is pertinent as research suggests that most individuals who smoke daily begin their use during adolescence and emerging adulthood (Perry et al., 2018). Further, young adults whosmoke intermittently or occasionally appear to maintain this behaviour over an extended duration and thus may require intervention (Doran et al., 2022). Therefore, detecting increased delay discounting in young people may provide the opportunity to target intervention resources more impactfully, potentially interrupting excessive smoking trajectories before they are initiated.

A major strength of the current study is that it comprises one of the largest samples to date in the investigation of delay discounting and smoking. Additionally, it is the first longitudinal study to examine the transition between smoking states to determine whether delay discounting differentiates individuals who transition from occasional to daily use. There are, however, some limitations that should be addressed. In the ESPAD questionnaire, smoking frequency is assessed via an ordinal question with seven categories, each encompassing a range of smoking frequencies. Responses were found to be highly skewed, and it is likely that the design of this question potentially obscured meaningful variation in smoking. To better capture the gradual escalation in smoking behaviour over time, we modelled our latent growth curve using frequency of use assessed via the TLFB. Notably, however, the TLFB was not administered at the second data collection wave, thus resulting in three waves for modelling. Nonetheless, three timepoints of data is the minimum required for latent growth curve modelling (Duncan and Duncan, 2009). Importantly, our model fit indices indicated that the model provided a good fit to the observed data. A further point of consideration is our classification of non-current use. We defined non-current use as individuals who had smoked previously but reported no use in the past 30 days. Therefore, this category included participants who ceased smoking following periods of either occasional use or daily use. Due to infrequent cases of participants transitioning from daily to non-current use, we opted to collapse both ex-occasional and ex-daily into one non-smoking category to ensure our multistate model derived robust estimates. Future longitudinal work should seek to include measures of smoking which enable better interrogation of differences between occasional and daily use, particularly with relation to smoking cessation. Despite these limitations, the current study provides an important first step in the longitudinal examination of transition from occasional to daily smoking.

To conclude, this study is the largest longitudinal investigation on the role of delay discounting in the initiation of smoking and the transition from occasional to daily use. Our findings indicated that delay discounting predicted the initiation of occasional smoking and the overall trend of smoking over time, but did not predict the transition from occasional to daily use. Further, delay discounting appeared to remain stable in the context of escalating smoking behaviour. Overall, these results suggest that delay discounting may serve as a risk factor for both the initiation of smoking and the trajectory of smoking, but that it may not necessarily differentiate between discrete states of smoking.

## Supplementary Material

1

## Figures and Tables

**Table 1:** Sample Characteristics at Four Waves (n = 1668).

	Wave 1	Wave 2	Wave 3	Wave 4

*N*	1400	1251	1224	1287
Age (years)	14.40 (0.40)	16.51 (0.59)	18.96 (0.73)	22.57 (0.68)
Sex (female)	723 (52 %)	653 (52 %)	656 (54 %)	690 (54 %)
MCQ *k*	−4.66 (1.49)	−4.65 (1.43)	−4.65 (1.42)	−4.88 (1.50)
Smoking status				
Never	1043 (75 %)	601 (48 %)	377 (31 %)	310 (24 %)
Non-current	236 (17 %)	317 (25 %)	374 (31 %)	503 (39 %)
Occasional	87 (6 %)	161 (13 %)	207 (17 %)	211 (16 %)
Daily	34 (2 %)	172 (14 %)	266 (22 %)	263 (20 %)
Number of cigarettes last 30 days (ESPAD)				
None	1279	918	751	813
Less than 1 cigarette per week	55	112	123	105
Less than 1 cigarette per day	32	49	84	106
1–5 cigarettes per day	17	96	102	95
6–10 cigarettes per day	7	49	92	96
11–20 cigarettes per day	4	22	57	58
More than 20 cigarettes per day	6	5	15	14
Number days smoked last 30 days (TLFB)^a^	0.59 (3.41)	-	6.15 (11.06)	6.24 (11.09)
Fagerström Test for Nicotine Dependence	0.03 (0.26)	0.20 (0.77)	0.37 (1.09)	0.38 (1.14)
AUDIT total score	1.31 (2.46)	3.79 (3.70)	5.87 (4.72)	6.12 (4.64)

*Note.* AUDIT = Alcohol Use Disorder Identification Test; ESPAD = European School Survey Project on Alcohol and Drugs; MCQ = Monetary Choice Questionnaire; TLFB = Timeline Follow-back

a TLFB was not acquired at wave 2

**Table 2:** State Transition Probabilities Over 8-year Period.

	Never	Non-current	Occasional	Daily

Baseline state				
Never	0.32 (0.29, 0.35)	0.34 (0.31, 0.36)	0.16 (0.15, 0.18)	0.18 (0.16, 0.20)
Non-current	0	0.44 (0.41, 0.48)	0.21 (0.19, 0.23)	0.35 (0.31, 0.38)
Occasional	0	0.44 (0.41, 0.47)	0.21 (0.19, 0.23)	0.35 (0.32, 0.39)
Daily	0	0.34 (0.29, 0.39)	0.16 (0.14, 0.19)	0.50 (0.43, 0.57)

*Note.* Row probabilities sum to 1. 95 % confidence intervals presented in parentheses.

**Table 3:** Hazard Ratios for the Effects of Delay Discounting, Age and Sex on Transitions Between Smoking States.

Transition	*In*(*k*)	Age	Sex

1 → 3	1.07 (1.02, 1.12)	0.82 (0.78, 0.86)	0.91 (0.78, 1.06)
2 → 3	1.59 (1.03, 2.46)	0.42 (0.22, 0.82)	0.25 (0.02, 2.68)
3 → 2	1.49 (0.96, 2.32)	0.46 (0.24, 0.89)	0.29 (0.03, 2.95)
3 → 4	1.09 (.99, 1.21)	0.79 (0.73, 0.85)	0.88 (0.66, 1.16)
4 → 3	1.02 (.87, 1.19)	0.99 (0.87, 1.11)	1.57 (1.06, 2.33)

*Note.* 1 = never, 2 = non-current, 3 = occasional, 4 = daily. *k* = discounting rate. 95 % confidence intervals presented in parentheses. Sex reference category = male.

## Data Availability

The data underlying the results presented in this study are available from the IMAGEN project (https://www.imagen-project.org/the-imagen-dataset). Data analysis scripts have been made publicly available online at the Open Science Framework (https://osf.io/mb75p/).

## Appendix A. Supporting information

Supplementary data associated with this article can be found in the online version at doi:10.1016/j.drugalcdep.2025.112955.
