# Supplementary material for: Trajectories of delay discounting and smoking from adolescence to young adulthood
Source: Drug Alcohol Depend. Author manuscript; Available in PMC 2026 Jul 26. (PMC13401516; doi:10.1016/j.drugalcdep.2025.112955)
Supplement: 1 [file NIHMS2185860-supplement-1.docx]

**Table 1S**

*Frequencies of Transitions Between Smoking States*

|  | To |  |  |  |
| --- | --- | --- | --- | --- |
| From | Never | Non-current | Occasional | Daily |
| Never | 1136 | 347 | 149 | 68 |
| Non-current | 0 | 488 | 155 | 122 |
| Occasional | 0 | 127 | 119 | 121 |
| Daily | 0 | 55 | 58 | 261 |

**Table 2S**

*Mean Sojourn Times*

|  |  | 95% CI | |
| --- | --- | --- | --- |
|  | Years | Lower | Upper |
| Never | 6.99 | 6.34 | 7.63 |
| Non-current | 0.16 | 0.02 | 1.48 |
| Occasional | 0.08 | 0.01 | 0.58 |
| Daily | 7.02 | 5.41 | 9.13 |

**Table 3S**

*Latent Growth Curve Model of Delay Discounting and Smoking Frequency*

|  | Estimate | *p*-value | Standardised  Estimate | CI lower | CI upper |
| --- | --- | --- | --- | --- | --- |
| **Free slope** | 5.23 | <.001 | 0.88 |  |  |
| **Regressions** |  |  |  |  |  |
| s_smoking ~ i_k | 0.11 | .002 | 0.67 | .21 | 1.13 |
| s_smoking ~ s_k | 2.36 | .005 | 2.04 | .51 | 3.56 |
| s_k ~ i_smoking | -0.48 | .369 | -1.90 | -6.69 | 2.89 |
| s_k ~ s_smoking | -4.09 | .347 | -4.73 | -14.28 | 4.83 |
| i_k ~ sex | -0.17 | .012 | -0.08 | ] |  |
| s_k ~ sex | -0.24 | .230 | -0.81 | -2.12 | 0.50 |
| i_smoking ~ sex | 0.01 | .798 | 0.01 | -0.08 | 0.10 |
| s_smoking ~ sex | 0.01 | .868 | 0.02 | -0.22 | 0.26 |
| **Covariances** |  |  |  |  |  |
| i_k ~~ i_smoking | .16 | <.001 | 0.24 | .16 | .33 |
| **Intercepts** |  |  |  |  |  |
| i_k | -4.34 | <.000 | -3.91 | -4.21 | -3.61 |
| s_k | 0.37 | .258 | 2.50 | -1.78 | 6.78 |
| i_smoking | -0.02 | .785 | -0.04 | -.33 | .25 |
| s_smoking | 0.53 | <.001 | 3.09 | 1.50 | 4.67 |
| **Variances** |  |  |  |  |  |
| k Baseline | 1.08 | .000 | 0.47 | .41 | .52 |
| k Fu1 | 0.94 | .000 | 0.46 | .42 | .50 |
| k Fu2 | 0.88 | .000 | 0.43 | .40 | .47 |
| k Fu3 | 0.49 | .000 | 0.22 | .13 | .31 |
| TLFB Baseline | 0.65 | .000 | 0.65 | .39 | .91 |
| TLFB Fu2 | 0.46 | .000 | 0.46 | .30 | .62 |
| TLFB Fu3 | 0.26 | .120 | 0.25 | -0.06 | .55 |
| i_k | 1.22 | .000 | 0.99 | 0.99 | 1.00 |
| s_k | 0.50 | .576 | 22.29 | -54.96 | 99.54 |
| i_smoking | 0.35 | .017 | 1.00 | 1.00 | 1.00 |
| s_smoking | 0.10 | .150 | 3.29 | -1.44 | 8.02 |

*Note.* i_smoking = intercept of smoking; s_smoking = slope of smoking; i_k = intercept of delay discounting; s_k = slope of delay discounting
